# Thirdhand Smoke and Neonatal/Pediatric Health: A Scoping Review on Nursing Implications

**DOI:** 10.3390/healthcare13243289

**Published:** 2025-12-15

**Authors:** Valentina Vanzi, Marzia Lommi, Alessandro Stievano, Gennaro Rocco, Maurizio Zega, Gabriele Caggianelli

**Affiliations:** 1 Center of Excellence for Nursing Scholarship (CECRI), Board of Nursing (OPI) of Rome, 00136 Rome, Italy; 2 JBI Italy Evidence-Based Practice and Health Research Centre, 00136 Rome, Italycaggianelligabriele@gmail.com (G.C.); 3 Department of Clinical and Molecular Medicine, Faculty of Medicine and Psychology, Sapienza University, 00189 Rome, Italy; 4 Department of Clinical and Experimental Medicine, University of Messina, 98125 Messina, Italy; 5 Department of Healthcare Professions, Azienda Ospedaliera Complesso Ospedaliero San Giovanni Addolorata, 00184 Rome, Italy

**Keywords:** thirdhand smoke, children, infant, neonatal intensive care unit, nursing, scoping review

## Abstract

**Background/Objectives**: Thirdhand smoke (THS), residual tobacco pollutants persisting on surfaces, dust, and fabrics, poses specific risks to infants and children, yet its implications for nursing remain underexplored. This scoping review mapped existing evidence on THS in neonatal and pediatric contexts and synthesized nursing implications, focusing on nurses’ knowledge, unintentional environmental contamination, and educational roles. **Methods**: Following JBI methodology and reported according to the Preferred Reporting Items for Systematic Reviews and Meta-Analyses guidelines, a three-step search was performed across MEDLINE, CINAHL, Scopus, Web of Science, Cochrane Library, Google Scholar, and OpenGrey. Studies were included if they addressed (1) nurses’ knowledge, beliefs, and attitudes toward THS-related risks in infants and children; (2) nurses’ contribution to unintentional environmental THS contamination; or (3) nurse-led educational or preventive interventions targeting parents or communities. **Results**: Among 563 records, 8 met inclusion criteria. Four investigated nurses’ awareness and perceptions, revealing limited understanding of THS despite recognition of its harmfulness. One study examined contamination, detecting nicotine residues on nurses’ fingers, suggesting possible in-hospital transmission. No nurse-led interventions specifically targeting THS were found, though broader smoke-exposure education programs showed benefits when supported by nursing staff. **Conclusions**: Evidence is scarce but underscores significant gaps in nurses’ knowledge, clinical guidance, and educational initiatives concerning THS. Strengthening nursing education and research is essential to mitigate THS exposure in neonatal and pediatric settings and enhance nurses’ preventive and advocacy roles.

## 1. Introduction

The adverse health effects of tobacco smoke on both smokers and nearby non-smokers, especially infants and children, are well established [1,2]. Although the harms of secondhand smoke (SHS) are well documented, thirdhand smoke (THS) remains under-recognized and current research increasingly addresses it [3]. The World Health Organization (WHO) identifies protecting children from tobacco smoke exposure as a global public health priority [4,5]. Global estimates suggest that roughly four in ten children are exposed to secondhand smoke (SHS), a major source of indoor air pollution [6]. This concern extends to THS, a related, often under-recognized residue that likely reaches an even larger number of infants and children. THS is tightly linked to SHS exposure yet is more difficult to detect and measure, further complicating prevention and mitigation efforts. Few studies have focused on identifying THS in pediatric and neonatal populations. In particular, Mahabee-Gittens et al. (2025) [7] conducted a prospective cohort study involving more than 1000 children aged ≤ 11 years and their parents in a U.S. children’s hospital, using hand nicotine as a marker of THS contamination and salivary cotinine as an indicator of overall tobacco smoke exposure. The results showed that nearly all children, including those classified as “protected,” with no smokers or vapers in the home, strict household smoking bans, and no SHS exposure in the previous week, had detectable levels of hand nicotine, underscoring the pervasiveness of THS [7].

Historically, THS was first described by Graham et al. in 1953, while decades later, a 1991 investigation detected nicotine residues in the settled dust of smokers’ households [8,9]. THS represents a relatively recent concept in environmental and public health [10], and it refers to the residual contamination that remains after SHS has dissipated from the air [11]. It comprises tobacco-derived gases and particles that sorb to indoor surfaces (carpets, furniture, walls, blankets, and toys), accumulate in household dust, and linger on smokers’ clothing, hair, and hands [12,13]. THS typically occurs at lower concentrations than SHS but persists on surfaces and in dust, sustaining long-duration exposure [11]. These contaminants can persist from minutes to months, re-emit into indoor air, undergo chemical transformations, and progressively accumulate long after a smoking episode [14,15]. In particular, residual nicotine on surfaces can become increasingly hazardous as it reacts with indoor oxidants, such as nitrous oxide, ozone, and formaldehyde, forming tobacco-specific nitrosamines with carcinogenic potential [16,17]. This mixture comprises numerous compounds also found in SHS and additional, newly identified chemicals that have been linked to carcinogenesis, teratogenicity, reproductive toxicity, and adverse cardiovascular, pulmonary, and neurodevelopmental outcomes [7,18,19,20,21,22]. Because THS is detectable in dust, air, and on surfaces, exposure can occur through multiple pathways [10]. Specifically, exposure occurs through dust ingestion, dermal absorption, and the inhalation of volatile components, placing infants and children at high risk due to their thinner skin, higher respiratory rates, and behaviors such as crawling, sucking, and hand-to-mouth contact [23]. Although research into the impact of THS on child health is ongoing [7], emerging evidence indicates markedly adverse effects across multiple target organs and a likely contribution to the development of a range of diseases, posing health risks from early life onward [21]. A large part of the research on nicotine and health has been carried out in relation to tobacco smoke. However, preclinical evidence from both in vitro and in vivo studies indicates that nicotine by itself can harm the nervous, respiratory, immune, and cardiovascular systems, particularly when exposure takes place during vulnerable phases of development [21].

By definition, hospitalized neonates and children represent a particularly vulnerable population, not only due to their developmental immaturity and intrinsic fragility but also because their health conditions often require multiple therapeutic and nursing interventions. In these settings, the interaction with healthcare professionals, especially nurses, who handle, reposition, and perform procedures on these patients, is frequent and pervasive. Globally, nurses represent the largest cadre of health professionals. They are central to health-system performance, carrying out a broad spectrum of functions, from health promotion and disease prevention to treatment and rehabilitation [24]. Accordingly, there is a need to identify and synthesize the evidence linking nursing care practices with exposure to, and adverse outcomes of, THS among neonates and children in hospital settings, in order to delineate both critical gaps and strategic opportunities for the nursing role in healthcare delivery, health promotion and education, and disease prevention. The specific aims were to (1) investigate nurses’ knowledge, beliefs, and attitudes regarding THS health risks for infants and children; (2) describe evidence on nurses’ contribution to unintentional environmental THS contamination (i.e., in NICU or PICU settings); and (3) map studies addressing nurses’ educational roles, interventions, and models to improve parental and community awareness of THS.

## 2. Materials and Methods

A scoping review was conducted to map the evidence on the impact of THS on neonatal and child health and to synthesize nursing implications, following JBI methodology for scoping reviews [25] and reported in accordance with the Preferred Reporting Items for Systematic Reviews and Meta-Analyses (PRISMA) flow chart [26,27]. In accordance with the JBI methodology for scoping reviews, the research question was structured using the Population–Concept–Context (PCC) framework, which provides a transparent and systematic approach to defining eligibility criteria and guiding evidence synthesis. Within the PCC framework, the population referred to neonatal and pediatric patients, ranging from premature infants to adolescents up to 18 years of age. This population (P) was selected due to its particular vulnerability to THS exposure, linked to developmental immaturity and behavioral patterns, like hand-to-mouth contact and crawling. The concept (C) focused on the impact of nursing care on THS. This concept was operationalized across three dimensions: (1) nurses’ knowledge, beliefs, and awareness of THS-related risks; (2) nurses’ potential contribution to environmental contamination in neonatal and pediatric care settings, and (3) nurse-led educational and preventive interventions aimed at parents, caregivers, and communities. The context (C) was limited to hospital settings, including NICU, PICU, and general pediatric wards. Restricting the context to healthcare environments allowed the review to capture the specific dynamics of nurse–patient interactions and organizational factors influencing THS exposure. Based on the PCC framework, studies were included if they addressed at least one review aim and related sub-question, and focused on neonates, infants, and children up to 18 years, and/or on nurses caring for these populations in hospital settings. Eligible studies explicitly examined THS and aligned with one of three dimensions. Studies were excluded if they involved adult-only populations or if they did not explicitly address THS, focusing instead on SHS or active smoking. Furthermore, for the educational aim, educational interventions not led or guided by nurses were excluded when nursing involvement was absent or peripheral. With regard to study design, primary empirical research of any design (qualitative, quantitative, mixed-methods, observational, quasi-experimental, and trials where available) and grey literature with sufficient methodological detail (i.e., technical reports and conference papers) were considered. Publications without primary empirical data, including editorials, commentaries, animal or in vitro studies, and reports limited to prenatal exposure without neonatal/postnatal outcomes were also ineligible. A three-step search strategy was applied. First, a limited search of MEDLINE (PubMed) and CINAHL (EBSCOhost) was performed to explore title/abstract terms and subject headings related to THS, pediatric health, and nursing. Second, a comprehensive search using all identified keywords and index terms was undertaken across MEDLINE (PubMed), CINAHL (EBSCOhost), Scopus (Elsevier), Web of Science (Clarivate), the Cochrane Library, and Google Scholar (for supplementary retrieval, Table S1). Third, reference lists of included studies were hand-searched. Grey literature sources included OpenGrey. No date limits were applied, and language was restricted to few languages. Specifically, eligible languages were limited to those covered by the authors’ proficiency: English, French, German, Spanish, and Portuguese. Studies in other languages were excluded due to resource constraints. The main keywords were “third-hand smoke,” “children,” “infant,” “neonatal intensive care unit,” and “nursing”. Boolean operators (NOT, AND, OR) were used to refine results. The PRISMA 2020 flow diagram was used to depict the study selection process, conducted in July 2025 [27]. Rayyan software (free version-essential tools) was used to support study screening, deduplication, and selection/inclusion processes. First, duplicate records were identified and removed. Second, titles and abstracts were screened independently by two authors. Third, the full texts of potentially eligible articles were assessed for inclusion. In the event of discrepancies between the two primary reviewers, disagreements were first discussed in a consensus meeting using predefined decision rules (population, concept, context alignment, and minimum data sufficiency). If consensus could not be reached, a third senior reviewer independently assessed the record and adjudicated the final decision. Data were then charted using a standardized extraction table capturing authors and year, country, study design and aim, nursing focus, and main findings. Each study was mapped to the scoping review framework to extract data addressing the specific objectives. The protocol was retrospectively registered on the Open Science Framework (OSF), and the registration can be viewed at: https://doi.org/10.17605/OSF.IO/U3BEH [28].

## 3. Results

**Figure 1:**
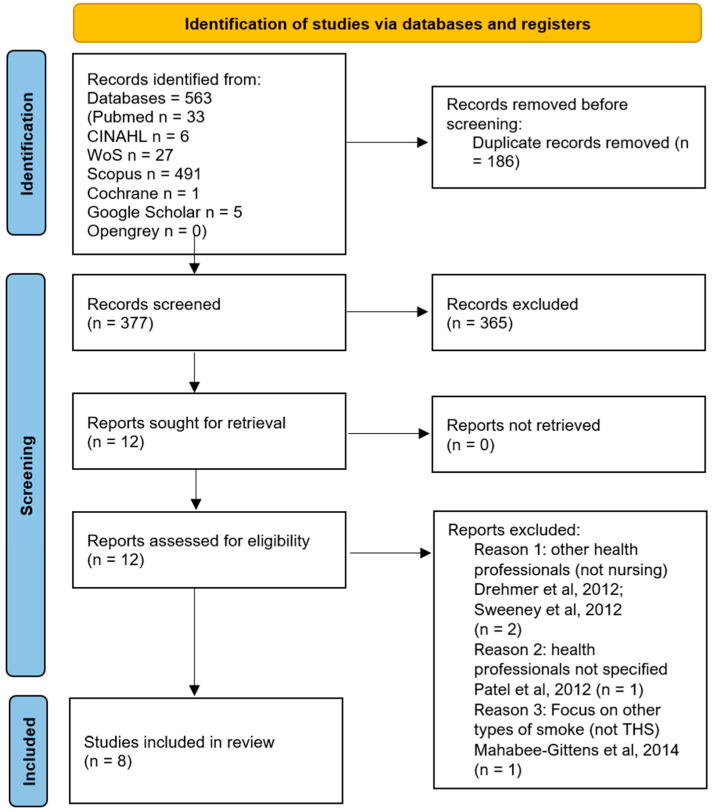
The PRISMA flow chart of the scoping review, [29,30,31,32].

The initial identification of potentially eligible studies through database searches yielded 563 records, reduced to 377 after removing 186 duplicates. Subsequently, following title and abstract screening, 12 full-text articles were assessed. This scoping review, as documented in the PRISMA flowchart, resulted in the final inclusion of eight articles (Figure 1). The selected studies were then categorized by thematic domain in relation to the project’s research question, enabling a targeted synthesis of evidence across the areas of nursing (i) knowledge and awareness, (ii) environmental contribution in neonatal/pediatric settings, and (iii) educational role, models, and interventions on THS.

### 3.1. First Sub-Question

For the first focus of this scoping review, four studies met the inclusion criteria: one meta-analysis [33] and three cross-sectional surveys [34,35,36]. No randomized controlled trials (RCTs) were identified. Although the search imposed no date limits, publications clustered in the past decade, with three of four (75%) appearing in the past three years. Two of four (50%) studies were conducted in Turkey [33,36]. Across studies examining health professionals’ and nurses’ knowledge, it was not possible to stratify findings with a specific focus on nurses but overall, prior awareness of THS ranged from 23.7% [36] to 35% [34], while 74.2% [35] to 89.8% [33] judged it harmful, especially for children. In a recent meta-synthesis, Yildirim-Ozturk et al. [33] estimated the overall prevalence of people’s knowledge that THS is harmful at 80.1%, with substantial variability across samples. Among health professionals, awareness that THS is harmful for children reached 89.8%, the highest prevalence reported in the meta-analysis. Notably, the review’s evidence base for health-professional perspectives drew on only three studies, focusing primarily on pediatric clinicians and hospitalists [29,37]. Only one included nurses among the participants [35]. In a recent cross-sectional study based on snowball sampling across Africa, the Americas, Asia, and Europe (*n* = 233), investigators explored health professionals’ knowledge and opinions regarding THS in a multinational context. The final sample, living in 24 different countries, was predominantly female non-smokers, held at least an undergraduate degree, and reported more than 15 years of experience [35]. Referring to the focus of this scoping review, two thirds (67.4%) were identified as nurses. Strikingly, before receiving a definition, nearly two in three participants (65.2%) had never heard of the term THS. Once explained, however, three quarters (74.2%) judged THS to be at least quite harmful to health, and almost nine in ten (89.3%) considered it at least quite harmful to children, indicating that unfamiliarity pertains largely to terminology rather than risk appraisal. Despite this, 76.7% believed that THS receives insufficient attention within their own healthcare settings, and roughly half estimated that their coworkers are not informed about THS. Notably, the proportion reporting prior awareness of THS, approximately one third, mirrors estimates from Darlow et al. [34], suggesting that limited baseline literacy may be widespread across jurisdictions and disciplines. Darlow et al. [34] conducted an online survey of healthcare professionals (*n* = 204) working at a cancer center and an affiliated general hospital to assess beliefs, attitudes, and health-education practices regarding THS, and to examine their associations with smoking-related attitudes and beliefs. The sample was predominantly nurses (113; 55.7%). About one third of respondents (35%) had heard of THS prior to completing the questionnaire, while more than two thirds (68.6%) believed that THS does not receive sufficient attention in clinical settings. These findings highlight limited baseline literacy and a perceived gap in institutional prioritization of THS, even among oncology-affiliated staff. The only study with a specific focus on nurses was recently published by Akdeniz et al. [36]. The research was conducted at a Turkish education and research hospital and included 219 nurses. This cross-sectional study used a questionnaire developed from a literature review together with the Thirdhand Smoke Awareness (THSA) scale. Only 23.7% of participants had previously heard of the THS concept, aligned with benchmarks from earlier studies, and the mean THSA score was 37.15 ± 7.34 [36].

### 3.2. Second Sub-Question

The second focus of this scoping review aimed to highlight the available evidence on the contribution of nurses to environmental THS contamination in neonatal and pediatric care contexts, whether through personal behaviors or clinical practices. Only one study met the inclusion criteria for this section. Northrup et al. [38] investigated potential contamination routes of THS in the NICU by using finger nicotine as a proxy measure. NICU medical staff completed a survey on smoking and electronic nicotine delivery system (ENDS) use/exposure and household characteristics. Then 35% healthcare professionals were randomly selected for a finger-nicotine wipe. Of 246 participating staff, nursing staff (*n* = 170; 65.6%) and nurse practitioners (*n* = 12; 4.6%) constituted the majority, followed by other medical staff. Over three quarters (78.5%) reported some exposure to tobacco smoke or ENDS vapor/aerosols. After field-blank adjustment, the median finger-nicotine level was 0.232 ng per wipe, and 78.3% of staff had measurable nicotine on their fingers. In adjusted models, proximity to smoking in friends’/family members’ homes and greater finger-surface area were significantly associated with elevated finger-nicotine levels (*p* < 0.05). Taken together, these findings suggest plausible transfer pathways of nicotine into the NICU environment via staff hands, with substantial nursing involvement simply by virtue of workforce composition, and underscore the need for targeted exposure-reduction policies and hygiene protocols in neonatal settings [38].

### 3.3. Third Sub-Question

Regarding the third evidence domain, mapping studies on nurses’ educational role, interventions, and models to improve parental and caregiver awareness of THS, the search did not identify studies with a specific focus on THS. Instead, existing work addresses tobacco smoke exposure (TSE) more broadly, encompassing both secondhand and thirdhand exposure. In this area, we found three studies that explicitly delineate nursing activities: one meta-synthesis, one qualitative study, and one quality-improvement project. Two (66.7%) were conducted in the USA and one in Australia. In terms of timing, all three were published within the past decade, a pattern consistent with the longer-standing research emphasis on SHS relative to the more recently articulated concept of THS. According to the meta-analysis conducted by Daly et al. [39], nurses were involved in 11 of the 16 included studies evaluating parent-focused counseling models on tobacco smoke exposure (TSE) within routine child healthcare. However, the pooled evidence did not demonstrate that interventions delivered by healthcare professionals in these settings reduced child TSE, increased parental smoking cessation, or lowered the number of cigarettes smoked. On the basis of this review, the effectiveness of routine child-health-based interventions delivered by health professionals, including those involving nurses, remains to be established. Merianos et al. [40] examined why evidence-based guidelines for addressing parental tobacco use and child tobacco smoke exposure (TSE) are suboptimally delivered in pediatric emergency department (PED) and urgent care (UC) settings, with the goal of informing strategies for consistent implementation. Using semi-structured, focused interviews with 29 actively practicing PED/UC professionals, 51% nurses, alongside physicians and administrators, the authors mapped current screening and counseling practices and explored perceived barriers and enablers. Participants commonly reported that, although they view intervening on parental tobacco use as part of their professional role, they do not currently follow guideline-concordant workflows. The principal barriers cited across roles were lack of knowledge, resources, and training specific to evidence-based tobacco counseling. Most respondents felt they were not skilled beyond “ask and advise,” attributing this limitation to inadequate training and the absence of practical supports. In a quality improvement project by Fergusson et al. [41], screening rates and the proportion of families receiving counseling about tobacco smoke exposure (TSE) increased markedly by the introduction of a nurse at the first-line TSE screening. The intervention assigned screening to nursing staff using a single, nurse-refined question, deliberately reworded to minimize parental offense, and embedded the screening tool within nursing records. This workflow change streamlined identification of TSE, normalized counseling as part of routine care, and contributed to sustained improvements in both screening and counseling delivery by clinicians. The included articles’ information and findings are comprehensively summarized in Table 1.

## 4. Discussion

A recent bibliometric analysis examined global trends in THS research, highlighting that despite increasing evidence of its health risks, scientific output remains limited and no public policies specifically address THS exposure. The analysis identified 227 publications indexed between 2009 and 2023, showing a steady annual growth of approximately 14% [42]. Referring to the first review domain, there is limited evidence related to knowledge, beliefs, and attitudes about the health risks of THS for infants and children among healthcare professionals [35], especially nurses. The concept of THS and recognizing its harmfulness represent crucial steps in preventing its adverse health impacts, and they underpin an awareness that can be translated into proactive, professional educational interventions to convey accurate information about risks and effective protective strategies for newborns and children. Publication timing shows growing scientific interest in this topic, with most papers appearing in recent years. A disproportionate share of studies originates from Turkey, which is emerging as a leading driver of the THS literature and demonstrates notable attention and sensitivity to the issue. Despite these loci of conduct, respondents spanned Africa, the Americas, Asia, and Europe, broadening external relevance and strengthening generalizability. Referring to the first sub-question of this scoping review, several issues stand out. First, there is a paucity of nurse-specific studies, despite nurses being the largest health workforce and having a wide intra- and extra-hospital presence that positions them to reach large populations and potentially broaden the dissemination of accurate THS information. With the exception of Akdeniz et al. [36], most studies employed multiprofessional samples, making it impossible to attribute responses specifically to nurses. Second, there is substantial heterogeneity in assessment tools used to measure THS knowledge and beliefs, which complicates cross-study comparison. In addition, the literature includes multiple investigations of these aspects; however, they are directed mainly at physicians, both staff and trainees, and medical students, with comparatively little focus on nursing and other health professionals [11,43,44,45]. Across all studies, respondents consistently noted that THS remains under-addressed in professional education and practice. These perceptions, aligned with the growing body of evidence on THS toxicity, point to a substantial and actionable gap in professional education [35]. The included studies’ authors’ conclusions converge on the same point: integrating THS content into continuing education, clinical guidelines, and health-promotion materials could help convert awareness into routine counseling, environmental risk-reduction, and family-centered protection strategies. There is a clear opportunity to embed this content throughout nursing education pathways, from entry-level courses through specialist training, promoting consistent uptake and practice. It is important to emphasize that building knowledge and awareness among health professionals, including nurses, is only the first step toward delivering accurate messages with appropriate educational strategies. Beyond theoretical preparation, practitioners need practical counseling skills to translate awareness into effective, goal-directed interventions. In this regard, Decker et al. [46] assessed pediatric RNs’ knowledge, attitudes, and behaviors regarding tobacco-cessation advice for parents who smoke. Of the nurses surveyed (*n* = 87; 67% response rate), only 22% reported routinely assessing parental smoking status and 14% encouraged parents to quit. Even fewer provided specific counseling or assistance. These findings underscore a persistent knowledge-to-practice gap and point to the need for targeted training, workflow supports, and reinforcement within continuing education to achieve consistent, effective parent-focused counseling.

Regarding the second sub-question on nurses’ potential contribution to environmental THS contamination in neonatal/pediatric care, evidence is limited to one study to date [38]. Nonetheless, the results highlighted a highly consequential phenomenon because almost four in five NICU staff had measurable finger nicotine, with finger surface area and frequency of reported exposure to tobacco smoke in friends’/family members’ homes emerging as important correlates [47]. Emerging work is beginning to examine clinical conditions and biomarkers in NICU infants, reflecting their extreme vulnerability. Notably, an observational study by Northrup et al. [48] investigated gut microbiome differences among NICU-admitted infants with varying levels of THS-related exposure. THS exposure was significantly associated with shifts in the infant gut microbiome, underscoring a plausible biological pathway through which THS may affect neonatal health in intensive care settings. Other studies underlined that THS, even in the absence of SHS, may drive microbiome dysbiosis with potential implications for childhood disease [49]. Moreover, evidence indicates that complete removal of nicotine residues is difficult to achieve, including on the fingertips of non-smoking medical personnel working in a smoke-free hospital [50,51]. Non-smoking policies are strictly enforced in hospital settings, like the NICU, but these environments may still become contaminated by nicotine and THS, as shown in previous work where incubators/cribs and other furniture had detectable surface nicotine [38,50]. For NICU infants whose caregivers or visitors smoke, THS can be carried into the unit on clothing, skin, and personal items, depositing on surfaces at concentrations comparable to those found in smoker households [38,50]. Given that many hospitalized neonates are immunocompromised and all are undergoing rapid lung development, no level of THS exposure is safe. Exposure should therefore be minimized as much as possible, with heightened vigilance during the critical early postnatal period, the first days, weeks, and months of life. Future research will determine the impact of THS on NICU infants [38].

The last sub-question, referring to the nurse-based educational strategies or models aimed at improving parental or community awareness of THS, was the most surprising. Public understanding of THS and its health risks, particularly for neonates and children, is still limited and poorly disseminated [52]. Despite the importance of education in tobacco control, to the authors’ knowledge, no studies have specifically examined the nursing role in parental counseling on THS. Existing work addresses tobacco smoke exposure (TSE) more broadly, encompassing both secondhand and thirdhand exposure, with parental smoking as the principal source of children’s exposure. Consequently, interventions have focused on promoting avoidance strategies, i.e., not smoking in the home, supporting parental smoking cessation, and preventing postpartum maternal relapse. Child healthcare services are a common and opportune setting for delivering parent-targeted interventions, as parents may be particularly receptive to advice during pediatric encounters. Nurses occupy a strategic frontline position in the health system, often serving as the first point of contact for families. This vantage point creates early opportunities to screen for tobacco use, initiate brief interventions with parents, and establish rapport that can be reinforced through ongoing therapeutic education. Such leverage is clear in Fergusson et al. [41], where nurse-led screening embedded in nursing records improved TSE screening and counseling delivery, and is echoed in other work on parent-focused interventions [30]. Current guidelines recommend that health professionals in child health settings incorporate routine, parent-focused counseling on TSE risk into standard care [53]. Beyond the literature’s limitation on nursing and counseling on THS, nurses play a crucial educational role in smoking cessation, and this statement is well supported by the scientific literature [54]. Several methods of delivering education have been tested, including distributing health education materials, providing face-to-face health education during hospitalization or telephone follow-ups after discharge, and organizing smoking cessation lectures or seminars [54]. These methodologies may also be tested and prove to be useful in THS parental counselling. Nurses have a unique opportunity to provide tobacco counseling to smoking and non-smoking parents in different settings, including during their child’s emergency department (ED) or hospital visit.

This scoping review lays the groundwork for practice and research implications by highlighting that THS is common in neonatal and pediatric settings, yet remains under-recognized and only marginally integrated into nursing practice. In clinical terms, findings underscore the need to strengthen nurses’ foundational literacy on THS, embed routine screening for tobacco smoke exposure in child health encounters, and normalize brief, parent-focused counseling as part of standard care. Integrating THS content into pre- and post-licensure curricula, continuing education, and clinical documentation may help translate awareness into consistent, family-centered protection strategies. At the same time, from a research perspective, the paucity of nurse-centered studies calls for intervention-based designs and longitudinal work explicitly focused on nursing roles. Priorities include mapping competencies, testing nurse-led educational and counseling programs, evaluating their impact on parental behaviors and child exposure, and further investigating nurses’ potential contribution to environmental contamination and its clinical consequences. Building this evidence base is essential to inform guidelines, support policy development, and enhance nurses’ preventive and advocacy roles in safeguarding infants and children from THS.

This scoping review has several limitations. First, the available evidence on THS in neonatal and pediatric contexts is extremely limited, with only eight studies meeting inclusion criteria and most addressing THS only indirectly or through multiprofessional samples rather than focusing specifically on nurses. As a result, the findings may not fully capture the breadth of nursing implications related to THS exposure. Second, heterogeneity in study designs, measurement tools, and operational definitions, particularly regarding THS knowledge, awareness, and environmental contamination, limits comparability across studies and precludes any form of quantitative synthesis. Third, this review restricted inclusion to a small set of languages due to resource and proficiency constraints, potentially excluding relevant evidence published in other languages. Fourth, grey literature was included but remains inherently variable in methodological quality, and no critical appraisal of individual sources was performed, in accordance with JBI scoping review guidelines. Finally, the lack of nurse-led interventional studies and the predominance of observational designs limit the ability to infer causal relationships or to draw practice-oriented conclusions. Despite these constraints, this review provides a valuable overview of an emerging and underexplored field, highlighting major evidence gaps and priorities for future research.

## 5. Conclusions

The World Health Organization recognizes tobacco use as a global epidemic and simultaneously designates the protection of children from tobacco smoke exposure as a key global public health priority. All tobacco products are harmful, and no level of exposure is safe. This scoping review highlighted that THS exposure in pediatric contexts is common, insufficiently recognized, and poorly integrated into nursing practice. Worldwide, nurses constitute the largest group of healthcare professionals and play a pivotal role in the functioning of health systems. Their responsibilities span a wide continuum of care, including health promotion, disease prevention, clinical management, and rehabilitative support. This scoping review specifically highlights nursing implications in safeguarding infant and child health with respect to THS, examining three thematic domains. Overall, the literature is sparse and rarely nurse-centric, with limited attention to the profession’s specific implications. Across studies, nurses’ baseline knowledge and awareness of THS tend to be low-to-moderate, broadly comparable to other health professions, yet respondents consistently recognize gaps in training and express interest in further education. Only one study directly delineates a plausible contamination pathway via nurses’ hands in neonatal intensive care settings. The greatest evidence gaps emerge around the educational role and the outcomes of nurse-delivered interventions. A recurring issue is the conflation of THS with SHS, which risks underrecognizing THS’s distinct mechanisms and impact. Evidence consistently shows that public understanding of THS and its health risks, particularly for neonates and children, is still limited and poorly disseminated [52]. To address this, research is needed that explicitly centers nursing: mapping competencies, testing targeted training, and evaluating counseling workflows and outcomes. Future studies should include intervention-based research, based on nurse-led educational and counseling programs, as well as longitudinal assessments of nurses’ awareness, attitudes, and practices related to THS, in order to capture changes over time and identify persistent gaps. Given nurses’ strategic numbers and reach across inpatient and community settings, structured educational pathways that strengthen foundational THS literacy, normalize parent-focused counseling, and include outcome evaluation could translate into meaningful protection for the youngest patients. Integrating THS content into pre- and post-licensure curricula and continuing professional education programs, together with purpose-built modules, embedded prompts in nursing records, and clear referral resources, may help convert awareness into consistent practice, ultimately reducing infants’ and children’s exposure to THS.

## Figures and Tables

**Table 1:** Characteristics of included studies focusing on nursing implications referring to THS and neonatal/pediatric health.

Authors, Year	Country	Study Design	Aim of the Study	Nursing Focus	Main Findings	Scoping Review’s Sub-Question
Daly et al., 2016 [39]	Australia	Meta-analysis	To determine the effectiveness of interventions delivered by healthcare professionals who provide routine child health care in reducing TSE in children (primary outcome)	Eleven of the sixteen trials in this meta-synthesis included nurses in the educational intervention/model provided	Interventions delivered by HCPs who provide routine child healthcare may be effective in preventing maternal smoking relapse. Further research is required to improve the effectiveness of such interventions in reducing child TSE and increasing parental smoking cessation.	Education/counseling
Darlow et al., 2017 [34]	USA	Cross-sectional study	To assess beliefs and behaviors regarding THS among healthcare professionals	Multiprofessional survey: the sample included 204 HCPs and around half of them were nurses (n. 113; 55.7%)	A little more than one third (35%) of the sample had heard of THS before completing the survey, and more than two thirds (69%) of the sample believed that THS issues do not receive enough attention.	Awareness/knowledge
Northrup et al., 2019 [38]	USA	Observational cross-sectional study	To explore contamination routes by characterizing nicotine levels (THS proxy) found on the fingers of NICU medical staff and to assess finger-nicotine correlates	Multiprofessional-based study: the final sample size was 246: the majority of participants were nursing staff (*n* = 170; 65.6%), the most numerous NICU specialty, and nurse practitioners (*n* = 12; 4.6%)	Almost four in five NICU staff had measurable finger nicotine, with finger surface area and frequency of reported exposure to tobacco smoke in friends’/family members’ homes emerging as important correlates.	Environment contribution
Ferguson et al., 2022 [41]	USA	Quality improvement project	To increase the rates of TSE screening and provider counseling regarding TSE reduction using an evidence-based approach	Multiprofessional project: role reconfiguration with nurse-led TSE screening	By integrating TSE screening into established nursing vital-signs records, the practice achieved sustained child TSE screening, with rates rising to 85%, surpassing the pre-specified 80% benchmark.	Education/counseling
Merianos et al., 2022 [40]	USA	Qualitative study	To use the theoretical domains framework (TDF) to identify current screening and counseling behaviors of PED/UC professionals related to parental tobacco use and child TSE, and determine barriers and enablers that influence these behaviors	Multiprofessional-based study: semi-structured interviews were conducted with 29 actively practicing PED/UC clinical staff; 51% were nurses	Most PED/UC professionals did not currently follow the guidelines, but perceived addressing parental tobacco use as part of their role. This study’s findings support the need to develop and implement an intervention to support PED/UC professionals in their tobacco prevention and control practices.	Education/counseling
Quispe-Cristóbal et al., 2022 [35]	Spain	Cross-sectional study	To explore the knowledge and opinions of healthcare professionals about THS	Multiprofessional survey: the final sample consisted of 233 participants and 67.4% were nurses	Almost two out of three HCPs who participated in the study did not know what THS was. Educational activities on this topic should be implemented.	Awareness/knowledge
Yildirim-Ozturk et al., 2024 [33]	Turkey	Meta-analysis	To determine the prevalence of people’s knowledge that THS is harmful for children and adults.	Of the twelve articles in this meta-synthesis, three targeted HCPs, but only one included nurses among the participants	For HCPs, the prevalence of people’s knowledge that THS is harmful to children was 89.8%, the highest prevalence value calculated in the meta-analysis	Awareness/knowledge
Akdeniz et al., 2025 [36]	Turkey	Cross-sectional study	To determine the awareness of THS among nurses	Nurse-based study: 219 nurses were included as participants	The study found that while the participants’ THSA was high, only a small proportion (23.7%) had heard of the concept. It is recommended to provide educational programs to inform nurses about THS.	Awareness/knowledge

Abbreviations: THS: Thirdhand smoke; TSE: Tobacco smoke exposure; ED: Emergency Department; HCP: Healthcare professional; NICU: Neonatal intensive care unit; PED: Pediatric emergency department; THSA: Thirdhand Smoke Awareness scale; UC: Urgent care.

## Data Availability

No new data were created or analyzed in this study.

## Supplementary Materials

The following supporting information can be downloaded at: https://www.mdpi.com/article/10.3390/healthcare13243289/s1.
