# Supplementary material for: Thirdhand Smoke and Neonatal/Pediatric Health: A Scoping Review on Nursing Implications
Source: Healthcare (Basel). 2025 Dec 15;13(24):3289. doi: 10.3390/healthcare13243289 (PMC12733024; doi:10.3390/healthcare13243289)
Supplement: Supplementary file 1 [file healthcare-13-03289-s001.zip › healthcare-3975995-supplementary.pdf]

## Supplementary Materials

**Table S1. Example of search strategy for MEDLINE (PubMed).**

| | |
|---|---|
| **#1** | infant* OR neonat* OR newborn* OR “premature infant” OR “preterm infant” OR children OR child OR paediatric* OR pediatric* OR “neonatal intensive care unit” OR NICU |
| **#2** | “third hand smoke” OR “third-hand smoke” OR “thirdhand smoke” OR THS |
| **#3** | nurs* OR “nursing staff” OR "nursing professional" OR "pediatric nurs*" OR “neonatal nurs*” OR "nurse practitioner" OR “health care professional” OR “healthcare professional” OR “health-care professional” |
| **Pubmed** | Search: ((infant* OR neonat* OR newborn* OR baby OR "premature infant" OR "preterm infant" OR children OR child OR paediatric* OR pediatric* OR "neonatal intensive care unit" OR NICU) AND ("third hand smoke" OR "third-hand smoke" OR "thirdhand smoke" OR THS)) AND (nurs* OR "nursing staff" OR "nursing professional" OR "pediatric nurs*" OR "neonatal nurs*" OR "nurse practitioner" OR "health care professional" OR "healthcare professional" OR "health-care professional") ("infant*"[All Fields] OR "neonat*"[All Fields] OR "newborn*"[All Fields] OR ("infant, newborn"[MeSH Terms] OR ("infant"[All Fields] AND "newborn"[All Fields]) OR "newborn infant"[All Fields] OR "baby"[All Fields] OR "infant"[MeSH Terms] OR "infant"[All Fields]) OR "premature infant"[All Fields] OR "preterm infant"[All Fields] OR ("child"[MeSH Terms] OR "child"[All Fields] OR "children"[All Fields] OR "child s"[All Fields] OR "children s"[All Fields] OR "childrens"[All Fields] OR "childs"[All Fields]) OR ("child"[MeSH Terms] OR "child"[All Fields] OR "children"[All Fields] OR "child s"[All Fields] OR "children s"[All Fields] OR "childrens"[All Fields] OR "childs"[All Fields]) OR "paediatric*"[All Fields] OR "pediatric*"[All Fields] OR "neonatal intensive care unit"[All Fields] OR ("intensive care units, neonatal"[MeSH Terms] OR ("intensive"[All Fields] AND "care"[All Fields] AND "units"[All Fields] AND "neonatal"[All Fields]) OR "neonatal intensive care units"[All Fields] OR "nicu"[All Fields])) AND ("third-hand smoke"[All Fields] OR "third-hand smoke"[All Fields] OR "thirdhand smoke"[All Fields] OR "THS"[All Fields]) AND ("nurs*"[All Fields] OR "nursing staff"[All Fields] OR "nursing professional"[All Fields] OR "pediatric nurs*"[All Fields] OR "neonatal nurs*"[All Fields] OR "nurse practitioner"[All Fields] OR "health-care professional"[All Fields] OR "healthcare professional"[All Fields] OR "health-care professional"[All Fields]) |
